# Phospholipid-Based Ultraflexible Nanovesicular Gel of Sertaconazole Nitrate for the Treatment of Skin Fungal Infections: Statistical Optimization, In Vitro and Preclinical Assessment

**DOI:** 10.3390/gels11110909

**Published:** 2025-11-13

**Authors:** Malleswara Rao Peram, Sachin R. Patil, Vidyadhara Suryadevara, Srinivasa Rao Yarguntla, Smita Kamalakar, Preeti Patil, Kamala Kumari Paravastu, Anand Vishwas Deshmukh, Manohar Kugaji, Sameer Nadaf

**Affiliations:** 1 Department of Pharmaceutics, Chebrolu Hanumaiah Institute of Pharmaceutical Sciences, Guntur 522019, Andhra Pradesh, India; svidyadhara@gmail.com; 2 Department of Pharmaceutics, Sarojini College of Pharmacy, Kolhapur 416004, Maharashtra, India; smitakamalakar3@gmail.com (S.K.); pbp.scop@gmail.com (P.P.); 3 Department of Pharmaceutics, Vignan Institute of Pharmaceutical Technology, Visakhapatnam 530049, Andhra Pradesh, India; ysrvignan@gmail.com (S.R.Y.); kamalaparavastu@gmail.com (K.K.P.); 4 Department of Pharmaceutics, KLE College of Pharmacy, KLE Academy of Higher Education and Research, Belagavi 590010, Karnataka, India; deshmukhanand7@gmail.com; 5 Central Research Laboratory, Maratha Mandal’s Nathajirao G. Halgekar Institute of Dental Sciences & Research Centre, Belagavi 590010, Karnataka, India; manoharkugaji@gmail.com; 6 Department of Pharmaceutics, Bharati Vidyapeeth College of Pharmacy, Palus 416310, Maharashtra, India; sam.nadaf@rediffmail.com

**Keywords:** sertaconazole nitrate, ultraflexible liposomes, nanovesicular gel, factorial design, antifungal agent

## Abstract

Sertaconazole nitrate (SN), a broad-spectrum antifungal agent, is clinically employed against diverse dermatophyte infections. Its therapeutic efficacy, however, is constrained by poor aqueous solubility (0.006 mg/mL) and insufficient skin penetration from current commercial formulations. To address these limitations, this research focused on developing, optimizing (using a 3^2^ factorial design), and assessing a topical nanovesicular gel incorporating sertaconazole nitrate-loaded ultraflexible liposomes (SN-UFLs) to enhance antifungal performance. The vesicles exhibited near-spherical morphology, with sizes ranging from 104.40 ± 1.20 to 151.90 ± 2.14 nm, zeta potential (ZP) values between −21.50 ± 1.25 and −51.20 ± 2.25 mV, and entrapment efficiency (EE) values from 77.60 ± 2.50% to 86.04 ± 3.20%. The optimized SN-UFL formulation (OPT-SN-UFL) was then integrated into a carbopol gel base. This SN-UFL-Gel was characterized for pH (6.5 ± 0.20), viscosity (499.66 ± 15 cP), spreadability (205 ± 1.50%), extrudability (154.18 ± 2.48 g/cm^2^), and drug content (96.7 ± 2.50%), as well as ex vivo skin permeation, skin irritation potential, and in vitro and in vivo antifungal efficacy. Compared with the marketed formulation, higher drug permeation and skin deposition were observed for SN-UFL-Gel. The SN-UFL-Gel exhibited a larger zone of inhibition (25 ± 1.50 mm) against *Candida albicans* compared to the commercially available formulation (20 ± 1.72 mm). The in vivo animal studies showed that SN-UFL-Gel showed better antifungal activity by efficient inhibition of infection induced in rats with *Trichophyton mentagrophytes*. The SN-UFL-Gel showed no signs of skin irritation and was stable at 4 ± 1, 25 ± 2, and 40 ± 2 °C for 3 months. Conclusively, the current work divulged successful augmentation of the overall effectiveness of sertaconazole nitrate by using deformable liposomes as a promising nanocarrier.

## 1. Introduction

A fungal infection, or mycosis, is a dermatological condition resulting from the invasion of tissues by fungi, leading to superficial, cutaneous, subcutaneous, or systemic infections. A significant number of people around the world suffer from fungal infections of the skin. Several factors, including the growing use of immunosuppressive drugs, heightened social congregation, and impaired immunity in patients with diseases like cancer, acquired immune deficiency syndrome (AIDS), and hematological disorders, are contributing to the alarming rise in fungal infections. Fungal infections of the skin, hair, and nails are extremely common, impacting almost one billion people globally [1]. Despite their prevalence, the social community deliberately ignores fungal infections. Typically, fungal infections begin on the skin’s surface and progress to penetrate deeper layers through the process of desquamation [2]. Cutaneous mycoses affect the *stratum corneum* or deeper layers of the epidermis, including its appendages (hair and nails), and elicit inflammation. It may be classified as dermatophytoses or dermatomycoses [3]. Dermatophytoses are an infection of the hair, skin, or nails caused by a dermatophyte, primarily by the *Trichophyton* genus and less frequently by the *Microsporum* or *Epidermophyton* genera, as dermatomycoses are caused by *Candida* species [4]. Topical antifungal medications are a cornerstone in the care of cutaneous mycoses [4]. Patients prefer topical antifungal therapies over systemic ones because they are non-invasive, simple to use, have direct access to the target site, avoid pre-systemic metabolism, reduce systemic toxicity, and improve the efficacy and bioavailability of the drug [5].

Sertaconazole nitrate (SN) is an effective topical broad-spectrum antifungal agent that belongs to the imidazole class. It is used in the treatment of various dermatophytic skin infections primarily caused by *Candida* species, *Trichophyton mentagrophytes*, *Trichophyton rubrum*, and *Epidermophyton floccosum* [6]. It is the treatment of choice for Athlete’s foot (tinea pedis), a form of ringworm infection affecting mainly the skin between the toes. It is also effective against skin and mucosal infections involving Gram-positive bacteria such as *Streptococci*, *Staphylococci*, *Listeria monocytogenes*, and *Trichomonas vaginalis* [7]. It acts through two distinct pathways. The primary mechanism of action is the inhibition of ergosterol production, a key component of the cell wall of fungi. Second, it causes intracellular component leakage when it directly binds to non-sterol lipids in fungal cell membranes at high concentrations. As a result, SN has fungicidal and fungistatic effects, depending on the dose [8]. In addition, SN has anti-inflammatory and antipruritic activities, which can reduce the edema, itching, severity of erythema, and formation of pustules associated with skin infections [9]. The anti-inflammatory properties of SN complement its antifungal activity, enhancing its effectiveness in treating various skin diseases. Because of its stronger fungistatic and fungicidal action against dermatophytes and yeasts, SN is being favored over other topical azoles such as bifonazole, fluconazole, miconazole, and clotrimazole [8].

Although sertaconazole nitrate (SN) demonstrates significant antifungal activity, its therapeutic utility is limited by poor aqueous solubility (0.006 mg/mL) and high lipophilicity (partition coefficient, log *p* = 6.23), resulting in inadequate skin permeation [10]. Currently marketed as a 2% topical cream for tinea pedis and other fungal infections, conventional formulations fail to achieve optimal healing rates. These systems primarily localize within the *stratum corneum* (the skin’s outermost layer) and cannot effectively deliver the drug to deeper infected tissues [10]. Consequently, advanced delivery systems capable of enhancing both skin penetration depth and drug deposition are essential to maximize SN’s therapeutic efficacy.

In recent times, there has been significant focus on nanovesicular carriers such as liposomes, niosomes, ethosomes, transfersomes, glycerosomes, and invasomes for the purpose of delivering drugs through topical and transdermal routes [11]. One such promising nanocarrier for enhanced penetration of drugs across the *stratum corneum* and targeting the drug molecules to the dermal region is the transfersome, also known as an ultraflexible, ultra-deformable, or elastic liposome. The components of transfersomes include phospholipids and surfactants, which act as edge activators. These edge activators allow vesicles to pass intact through the extremely tiny skin pores by providing the required flexibility of the lipid bilayer membrane [12].

Various formulations, including nanomicelles [13], microsponges [14], anhydrous gels [15], nanostructured lipid carriers [16], thermosensitive hydrogels [17], glycerosomes [10], liposomes [18], and leciplex of SN [19] have been reported in the literature. Mandlik et al. (2019) [20] developed and optimized SN-embedded flexisomes to enhance their cutaneous antifungal activity. However, the antifungal activity of SN flexisomes was not tested in animal models [20]. Abdellatif et al. (2017) formulated different lipid vesicles, such as liposomes, glycerosomes, ethosomes, and transfersomes of SN, and compared their antifungal efficacy [21]. Nevertheless, no optimization studies have been conducted for various formulation variables that impact the characteristics of lipid vesicles. The present study addresses these limitations by combining the statistically designed factorial design approach with comprehensive in vitro and in vivo evaluations. This approach will enable us to understand how critical formulation variables, such as phospholipid and sodium cholate concentration, affect the vesicle size and entrapment efficiency. In comparison to previously reported studies, this optimization establishes a rational basis for the development of an ultraflexible nanovesicular gel that exhibits improved antifungal efficacy and enhanced clinical relevance.

Given these considerations, this investigation sought to formulate, characterize, and optimize sertaconazole nitrate-loaded ultraflexible liposomes (SN-UFLs) to enhance the antifungal efficacy of SN. A 3^2^ factorial design was employed to evaluate the impact of formulation variables on SN-UFL properties and identify the optimal formulation. The optimized SN-UFLs were incorporated into a carbopol gel matrix and characterized for pH, rheological behavior, in vitro skin penetration, ex vivo permeation and drug deposition, histopathological effects, and in vitro antifungal activity. The antifungal performance of the resulting SN-UFL gel was subsequently evaluated against a commercial formulation using a relevant in vivo model.

## 2. Results and Discussion

### 2.1. Preparation and Optimization of SN-UFL

Sertaconazole nitrate-loaded ultraflexible liposomes (SN-UFLs) were formulated via the thin-film hydration-sonication technique [22]. A 3^2^ factorial design (Design-Expert^®^ v7.0.0) was employed for systematic optimization, with phospholipid concentration (Phospholipon^®^ 90G; X_1_: 750–950 mg) and sodium cholate concentration (X_2_: 50–250 mg) selected as independent variables following preliminary screening studies [23]. Vesicle size (Y_1_) and entrapment efficiency (Y_2_) were critical response variables. Nine experimental formulations were prepared, with physicochemical characterization data summarized in Table 1. Statistical analysis evaluated linear, quadratic, and two-factor interaction models using Design-Expert^®^ software, with model selection based on multiple criteria: coefficient of correlation (R^2^), adjusted R^2^, predicted R^2^, % coefficient of variation (CV), predicted residual sum of squares (PRESS), and adequate precision. Quadratic models demonstrated optimal fit for both responses (Table 2), confirmed by high R^2^ values (>0.95) and low %CV (<5%), indicating robust predictability.

### 2.2. Effect of Independent Variables on the Vesicle Size (Y_1_)

Vesicle size critically influences the delivery efficacy of nanovesicular systems designed for topical/transdermal applications, with smaller vesicles (<300 nm) demonstrating enhanced penetration compared to larger counterparts in multiple studies [24,25]. The SN-UFLs exhibited nanoscale dimensions (104.40 ± 1.15 to 151.90 ± 3.74 nm; Table 1), aligning with optimal penetration parameters. Statistical analysis confirmed a quadratic model (R^2^ > 0.98) as the most appropriate descriptor for the relationship between independent variables and vesicle size, with the equation:

Vesicle size (Y_1_) = +121.70 + 16.82 X_1_ − 7.37 X_2_ + 0.27 X_1_X_2_ + 8.45X_1_^2^ − 1.60 X_2_^2^ (1)

The quadratic model revealed a positive linear coefficient for phospholipid concentration (X_1_; +16.82) and a negative linear coefficient for sodium cholate concentration (X_2_; −7.37), indicating that vesicle size increases with higher Phospholipon^®^ 90G content but decreases with elevated sodium cholate levels [22]. The magnitude of X_1_’s coefficient (+16.82 vs. X_2_ − 7.37) demonstrates phospholipid concentration exerts a dominant influence on vesicle size relative to the sodium cholate component. ANOVA results (Table 3) confirmed model significance (F-value = 135.32; *p* < 0.0001), with X_1_ and X_2_ identified as statistically significant (*p* < 0.05). The high concordance between predicted R^2^ (0.9474) and adjusted R^2^ (0.9882) confirms robust model predictability. An adequate precision ratio of 33.07 (substantially exceeding the minimum threshold of 4) indicates an excellent signal-to-noise ratio, validating this model’s utility for defining the formulation design space in SN-UFL development.

**Figure 1:**
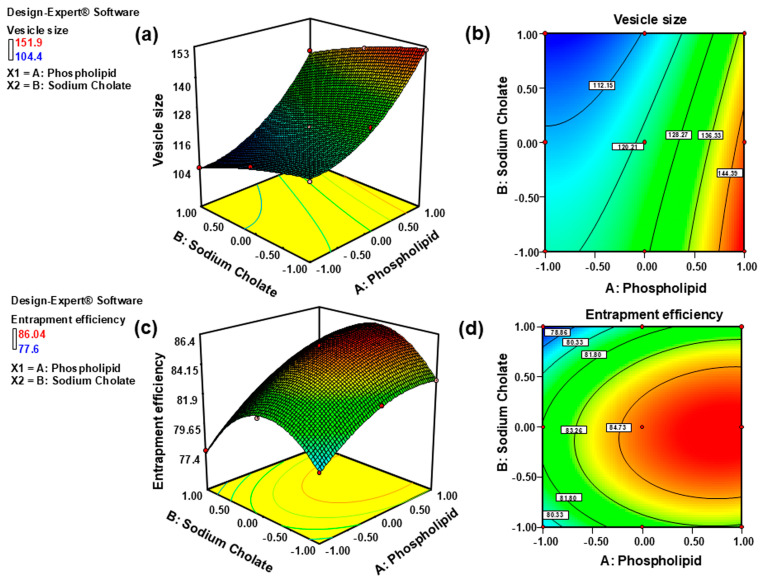
Three-dimensional (3D) response surface plots (**a**,**c**) and two-dimensional (2D) contour (**b**,**d**) plots showing the effect of independent variables on the vesicle size and entrapment efficiency of SN-UFLs.

Figure 1 (3D response surface and 2D contour plots) corroborates the inverse relationship between sodium cholate concentration and vesicle size and the direct correlation with phospholipid content. As phospholipid concentration increased from 750 to 950 mg, vesicle size expanded proportionally due to enhanced bilayer thickness and membrane rigidity [26]. Conversely, sodium cholate concentrations spanning 50–150 mg demonstrated a concentration-dependent size reduction, attributable to sodium cholate-induced interfacial tension reduction at the lipid-water interface. At concentrations exceeding 150 mg, sodium cholate promoted micellar transition, generating sub-50 nm mixed micelles rather than vesicular structures [27]. These observations align with established mechanisms in flexible lipid nanocarriers, consistent with prior reports on cilnidipine-loaded transfersomes where phospholipid concentration positively correlated with vesicle dimensions and sodium cholate exerted a size-reducing effect [28].

### 2.3. Effect of Independent Variables on the % EE (Y_2_)

The primary goal in the development of any nanovesicular drug delivery system is to produce vesicles with a high % EE of the drug. The % EEs of all formulations were found to be in the range of 77.6 ± 0.74 to 86.04 ± 2.75%. The effect of independent factors on the % EE of SN-UFLs was analyzed by following the quadratic equation.

% EE (Y_2_) = + 85.28 + 2.14 X_1_ − 0.69 X_2_ + 0.36 X_1_X_2_ − 1.26 X_1_^2^ − 3.43 X_2_^2^ (2)

The equation demonstrated that the percentage of EE is positively influenced by the quantity of phospholipid (X_1_) and negatively influenced by the amount of sodium cholate (X_2_). This suggests that the % EE of SN-UFL vesicles increased with increasing phospholipid concentrations but decreased with increasing sodium cholate concentrations [23]. The high magnitude of the coefficient X_1_ indicates that the quantity of phospholipid substantially impacts the % EE, as opposed to the concentration of sodium cholate. ANOVA results for entrapment efficiency (% EE) in Table 3 demonstrate model significance (F-value = 96.56; *p* < 0.0001). Statistically significant terms were identified at *p* < 0.05, confirming the model’s reliability in predicting % EE within the experimental design space. The model terms X_1_, X_2_, X_1_^2^, and X_2_^2^ are considered statistically significant. The predicted R^2^ of 0.9440 is in reasonable agreement with the adjusted R^2^ of 0.9835, signifying a good fit. Adequate precision assesses the signal-to-noise ratio, which is preferred to be greater than 4. The ratio of 31.08 suggests an adequate signal, indicating that this model can be utilized to guide the design space.

Figure 1 (3D response surface and 2D contour plots) reveals a concentration-dependent relationship between independent variables and entrapment efficiency (% EE). Phospholipid content (750–950 mg) demonstrated a linear positive correlation with % EE, attributable to increased lipid solubility and enhanced bilayer rigidity that facilitates SN incorporation [28]. Sodium cholate exhibited a biphasic effect: % EE increased from 50 to 150 mg due to edge activation, where surfactant integration into the phospholipid bilayer improved membrane fluidity and drug partitioning. Beyond 150 mg, % EE declined at 250 mg due to membrane destabilization characterized by pore formation and transition to mixed micelles (<50 nm) [29]. These micelles exhibit inherently low entrapment capacity owing to their high curvature, dynamic structure, and minimal hydrophobic core volume, as established in surfactant-lipid thermodynamics [30].

### 2.4. Selection of Optimized SN-UFL (OPT-SN-UFL)

**Figure 2:**
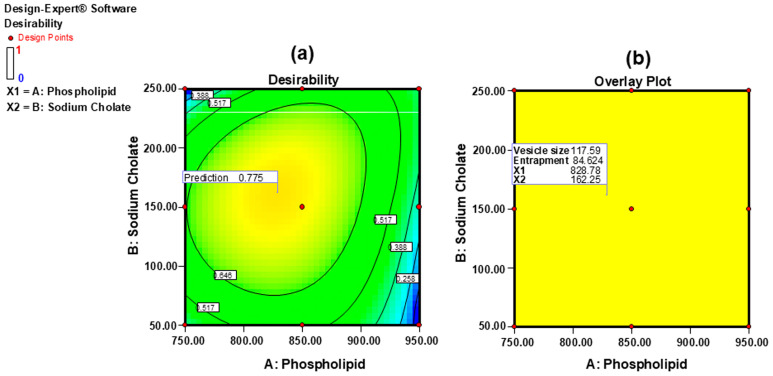
Selection of optimized formulation from Design Expert^®^ software: (**a**) desirability plot and (**b**) overlay plot.

The optimized transfersomal formulation was selected using the numerical optimization method available in Design Expert 7 software, focusing on achieving the highest desirability value. The optimization involved applying constraints to the independent variables, specifically minimizing vesicle size and maximizing entrapment efficiency [26]. The OPT-SN-UFL was achieved with a phospholipid 90G concentration of 828.78 mg and a sodium cholate concentration of 162.25 mg, with a desirability value of 0.775 (Figure 2). The predicted vesicle size (117.59 nm) and % EE (84.62) closely matched the actual values (vesicle size 118.60 ± 2.80 nm and % EE 84.10 ± 2.50), thereby reinforcing the reliability of the optimization process used in the preparation of SN-UFLs.

### 2.5. Size Distribution and Zeta Potential

**Figure 3:**
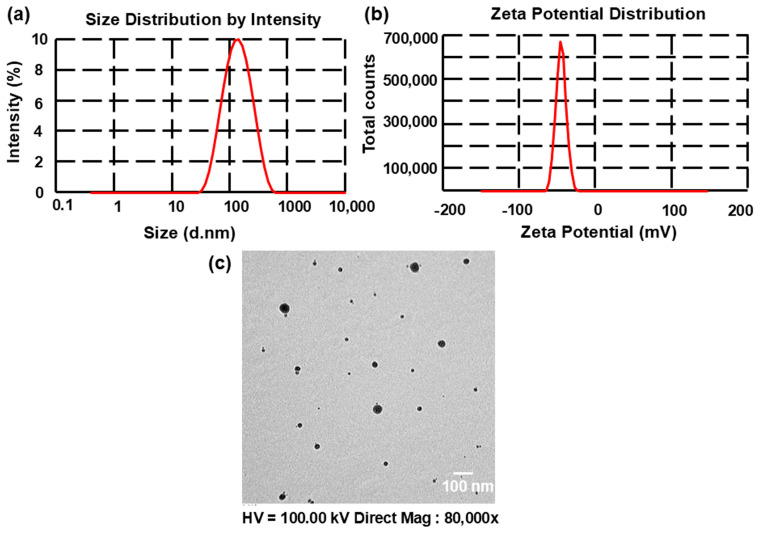
Vesicle size (**a**), zeta potential (**b**), and TEM image (**c**) of OPT-SN-UFL.

The SN-UFL’s size distribution profile was predicted by determining the polydispersity index (PDI) value. Values near 0 indicate a uniform size distribution, while values near 1 indicate a heterogeneous size distribution [31]. The PDI values of the formulated SN-UFLs ranged from 0.15 ± 0.01 to 0.28 ± 0.02 (Table 1), indicating a uniform vesicle distribution. Zeta potential is a reflection of the surface charge of the vesicles, which affects their stability, aggregation, and interaction with biological membranes. A higher ZP value increases electrostatic repulsion, reducing vesicle coalescence and ensuring a stable dispersion for effective drug delivery. The ZP values for the prepared SN-UFLs were found to be in the range of −21.5 ± 1.45 to −51.2 ± 2.45 mV, indicating their optimum stability [31] (Table 1). The negative charge of the transfersomes may be attributed to sodium cholate, an anionic single-chain surfactant utilized in their manufacture to enhance the flexibility [32]. These results match the findings of Ali et al. (2015), which indicated that the ZP of nano-transfersomes containing papaverine hydrochloride was −33.4 mV [33]. The vesicle size, PDI, and ZP of OPT-SN-UFL were found to be 118.60 ± 2.82 nm, 0.23 ± 0.02, and −35.20 ± 2.50 mV, respectively (Figure 3).

### 2.6. Vesicle Morphology

Transmission electron microscopy was used to observe the morphology of the OPT-SN-UFL (Figure 3c). The prepared vesicles were found to be spherical, discrete, and in the nano size range. These results coincide with those obtained by other researchers [34].

### 2.7. Preparation of SN-UFL Gel

In order to improve the ease of application and enhance the stability and controlled release of the active ingredients, the OPT-SN-UFL was incorporated into gel using Carbopol polymer. Carbopol^®^ 980NF was selected as the gel-forming polymer due to its non-irritant nature, superb bio-adhesion properties, and ability to form transparent gels [35].

### 2.8. Characterization of SN-UFL-Gel

The SN-UFL gel was white and opaque to translucent in appearance, had a smooth texture, had good homogeneity, and was free from gritty particles and phase separation. The most crucial criterion for formulations meant for topical or transdermal administration is their pH. Formulations with extremely acidic or basic properties might disrupt the skin’s natural pH balance and lead to discomfort. The pH of the SN-UFL gel was found to be 6.5 ± 0.20, which is within the physiological skin pH range, suggesting that the developed gel was safe, non-irritating, and appropriate for topical application [36]. Administering the gel in thin layers to the skin is a crucial attribute of its consistency. The viscosity of the gel indicates its consistency and significantly influences drug permeation. The viscosity of the SN-UFL gel was found to be 499.66 ± 15 cP (50 rpm and 25 °C), demonstrating that the gel possesses excellent consistency [37]. An optimal topical formulation should exhibit excellent spreadability and require minimal time for application. The term “spreadability” denotes the extent of the area over which gel disperses upon application to the skin or affected region. The spreadability is crucial for patient adherence and facilitates uniform application of the gel to the skin. Additionally, it affects the therapeutic efficacy of the gel. The spreadability of prepared gel was found in the range of 205 ± 1.50%, showing that the gel could be easily spread with minimal shear force [22,23]. The extrusion of the nanogels from the tube is necessary during its use and for patient adherence. The extrudability of the SN-UFL gel was found to be 154.18 ± 2.54 g/cm^2^, indicating an excellent extrudability property of the gel [38]. The drug content measurements were conducted to evaluate the homogeneous distribution of SN throughout the gel. The amount of drug in the SN-UFL gel was found to be 96.70 ± 0.20%. This means that the SN was distributed evenly in the carbopol gels and that almost no drug was lost during the formulation process [26].

### 2.9. Ex Vivo Skin Permeation and Deposition Studies

**Figure 4:**
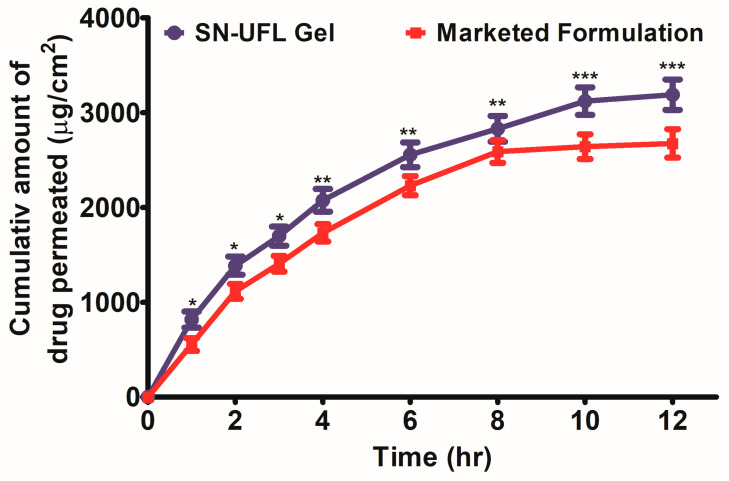
Ex vivo skin permeation studies of SN-UFL-Gel and marketed formulation through rat abdominal skin. Data are presented as mean ± SD (n = 6). * *p* < 0.05, ** *p* < 0.01, *** *p* < 0.001 vs. marketed formulation (unpaired *t*-test).

Ex vivo permeation studies are essential to better understand how pharmaceutical formulations designed for topical or transdermal drug delivery perform in vivo. In order to determine the formulation’s efficacy and reliability, these investigations use either animal skin or human skin that is relevant to the application site [39]. SN is a highly lipophilic compound (log *p* value 6.23) with poor aqueous solubility (0.006 mg/mL). Hence, in the present study, 50% *v*/*v* ethanol was added to the receptor medium to maintain the sink conditions and to allow better release of SN into the receptor medium. Without sufficient solubility in the receptor fluid, the drug concentration at the dermal side of the membrane would increase, thereby reducing the concentration gradient and decreasing its permeation. The literature confirms that 50% ethanol (*v*/*v*) in the receptor compartment did not affect drug molecule penetration into/through the skin or the skin barrier characteristics [40]. However, to better mimic the physiological state while still maintaining adequate sink conditions, further studies with alternative receptor media, such as PBS with lower ethanol concentrations or surfactant-based systems, should be explored. The permeation of SN from SN-UFL gel and a commercial formulation (Onabet^®^ Cream) through the abdominal skin of rats was evaluated using a Franz diffusion cell. Figure 4 displays the cumulative amount of the drug permeated versus time for the SN-UFL gel and the marketed formulation. The cumulative amount of SN permeated from SN-UFL gel (3188.57 ± 7.56 µg/cm^2^) was significantly higher (*p* < 0.001) compared to the marketed formulation (2674.28 ± 5.47 µg/cm^2^) following 12 h permeation. This substantial difference indicates that the SN-UFL gel was more effective in delivering SN through the skin over time. The SN permeation flux from SN-UFL (150.12 ± 0.97 µg/cm^2^/h) was significantly greater (*p* < 0.001) and 1.19 times higher than the marketed formulation (125.90 ± 0.85 µg/cm^2^/h). Further, the permeability coefficient values for SN-UFL and the marketed formulation were found to be 7.50 × 10^−3^ and 6.29 × 10^−3^, respectively. The enhanced permeation and flux of SN in SN-UFL gel may be attributed to several mechanisms associated with ultraflexible liposomal systems. Firstly, the vesicles are able to penetrate deeper layers of the skin due to their nano size and ultra deformability, which enable them to pass through the *stratum corneum*’s tiny intercellular spaces [41]. Second, in order to achieve enhanced vesicle penetration, the edge activators in transfersomes interact with skin lipids, which disrupts the *stratum corneum*’s tight lipid structure [42]. Further, when applied to the skin, the transdermal hydration gradient moves transfersomes from the *stratum corneum*, which is dehydrated (10–20% water), towards the hydrated epidermal layers underneath (65–70% water), allowing for deeper penetration [43]. These results are in agreement with previous reports where *althaea officinalis*-loaded transliposome (AO-TL) gel has demonstrated significantly improved permeability compared to conventional gel [44].

The effectiveness of topical antifungal therapy largely depends on the ability of the drug to accumulate within the skin layers, a property quantified by the local accumulation efficacy (LAC) [10]. In the present study, SN-UFL gel demonstrated significantly higher (*p* < 0.01) LAC values (4.49 ± 0.30) compared to the commercial formulation (2.10 ± 0.50). This indicates that incorporation of ultraflexible liposomes into the hydrogel matrix enhanced the local skin accumulation of SN by approximately 2.13-fold relative to the marketed product. The higher drug accumulation may be attributed to the enhanced interaction of SN-UFL gel with skin layers, resulting in the formation of greater drug depots from which the drug could be released into deeper layers [10]. These findings are consistent with those of Ma et al., who reported higher LAC values for transethosomes and ethosomes compared to commercial cream [45].

### 2.10. Skin Penetration Study

**Figure 5:**
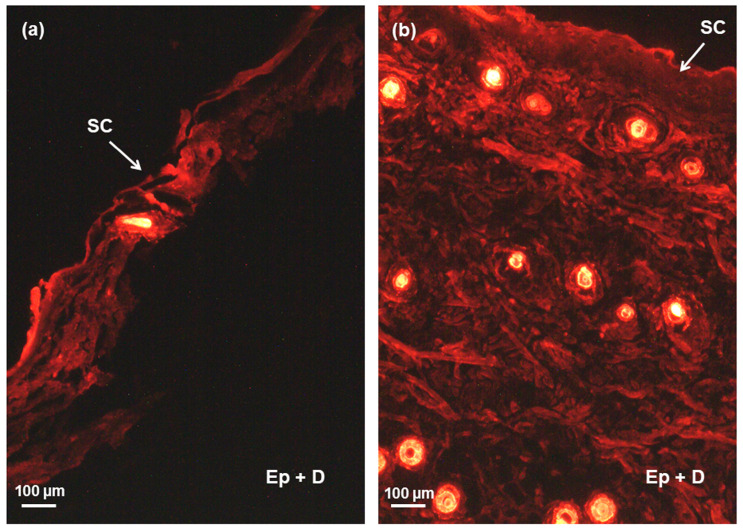
Fluorescence microscopic images showing the skin penetration ability of (**a**) Rh6G-Gel and (**b**) Rh6G-TFS-Gel. SC—*stratum corneum*, Ep + D—epidermis and dermis. Scale bar 100 µm.

The enhanced skin penetration potential of developed nanovesicular systems was investigated by employing a fluorescence microscope and lipophilic fluorescence dye rhodamine-6G (Rh6G). Rh6G, a red dye with an oil/water partition coefficient of 3.7, was encapsulated in the lipid bilayers of transfersomes to simulate the behavior of a lipophilic drug like SN [46]. Fluorescence photographs of the rat skin after 8 h treatment with Rh6G-Gel and Rh6G-TFS-Gel are shown in Figure 5. The fluorescence microscopic analysis demonstrated notable differences in skin penetration between the Rh6G-TFS-Gel and the plain rhodamine-6G gel. In the control group (plain Rh6G-gel), fluorescence was mostly limited to the *stratum corneum*, with very little penetration into the deeper epidermal and dermal layers. This suggests that the limited permeation capacity of the dye occurs when applied without a carrier system. In contrast, skin sections treated with Rh6G-TFS-Gel showed strong and uniform fluorescence intensity across the epidermal layer and into the dermis. A quantitative examination of fluorescence was conducted using ImageJ software (Version 1.54 g), which further confirmed that the Rh6G-TFS-Gel exhibited a higher fluorescence intensity (64.91 a.u.) in comparison to the Rh6G-Gel (44.05 a.u.). These results support the qualitative observation of deeper and more extensive fluorescence distribution in the skin layers with Rh6G-TFS-Gel, indicating enhanced penetration. The increased fluorescence intensity observed in deeper layers indicates the enhanced penetration capability of transfersomes. The enhanced penetration is due to the highly deformable and flexible bilayer structure of transfersomes, allowing them to navigate the narrow intercellular pathways of the *stratum corneum*. Further, the hydration gradient across the skin facilitates their penetration by serving as the driving force for vesicle movement [47]. The results indicated that transfersomal gel enhanced the penetration of the hydrophilic fluorescent probe, like Rh6G, and facilitated its deposition in the deeper skin layers. This property is advantageous for both topical and transdermal drug delivery by enhancing local bioavailability and therapeutic efficacy through deeper accumulation. These results are consistent with previous studies that demonstrated that transfersomes function better than conventional drug delivery systems in terms of skin penetration and drug deposition [41,44,48].

### 2.11. Skin Irritation Studies

**Figure 6:**
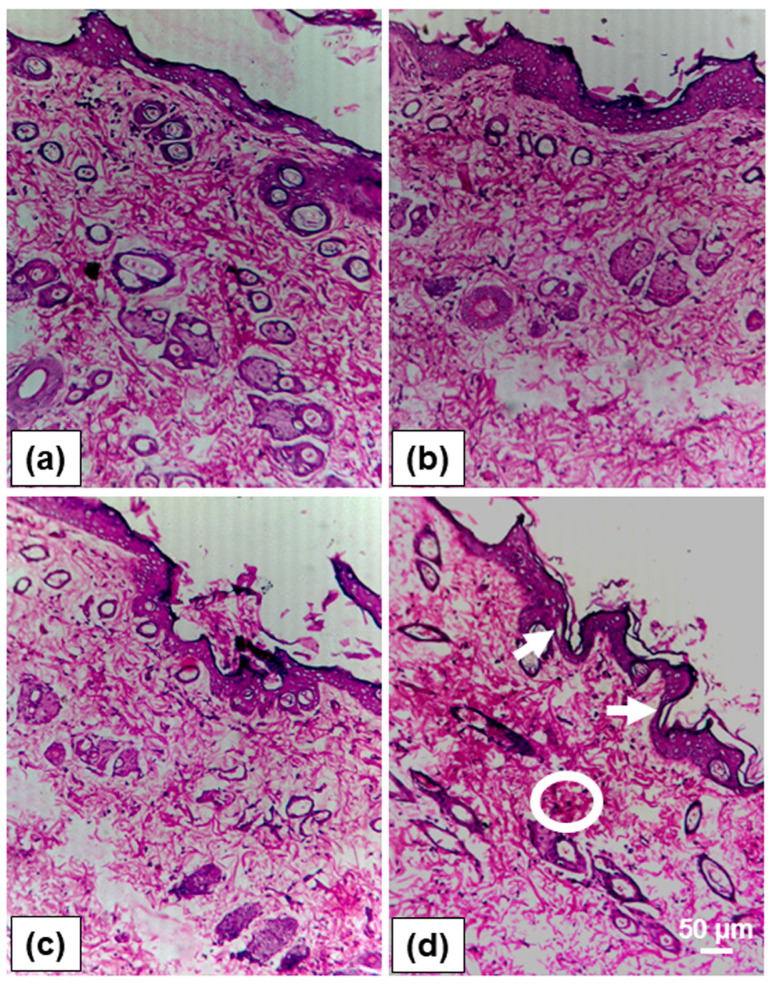
Histopathological photographs of skin treated with (**a**) control, (**b**) Blank–UFL gel, (**c**) SN-UFL-Gel, and (**d**) marketed formulation. Circles indicate the mild inflammatory cell infiltration, and white arrows indicate occasional focal disruption of the *stratum corneum*. Scale bar 50 µm.

One of the primary concerns with the topical and transdermal drug delivery system is safety to the skin from the developed nanovesicular formulation, which can be assessed through histopathology studies [49]. Histopathological evaluation was carried out on the rat skin to observe the effect of nanovesicular gels on the skin structures following their application. Throughout the seven-day study period, there were no visible signs of erythema, edema, or other aberrant skin reactions observed in any of the groups treated with PBS (control), blank-UFL-Gel, and SN-UFL-Gel. Conversely, few animals in the marketed formulation group exhibited extremely moderate erythema on day 3, which disappeared by the end of the study period. The histopathological investigation of rat skin treated with various formulations, including the PBS (control), Blank-UFL-Gel, SN-UFL-Gel, and the marketed formulation, is shown in Figure 6. The groups treated with PBS, Blank-UFL-Gel, and SN-UFL-Gel exhibited normal structural integrity of the epidermis and dermis, characterized by an intact *stratum corneum*, viable epidermis, and undamaged dermal tissue. There was no indication of cellular infiltration, epidermal thickening, or edema in these groups. In contrast, in the marketed formulation group, occasional focal disruptions of the *stratum corneum* and mild inflammatory cell infiltration in the dermis were observed, correlating with the temporary erythema noted in the visual observation. These results show that neither Blank-UFL-Gel nor SN-UFL-Gel caused any irritation or changes to the histology of rat skin, and both were well tolerated after repeated topical treatment. The lack of inflammatory alterations indicates that the developed formulations are safe for topical use. Similar results have been reported in the literature where nano-vesicular gels showed superior skin compatibility compared to conventional marketed products [49,50].

### 2.12. In Vitro Antifungal Study

**Figure 7:**
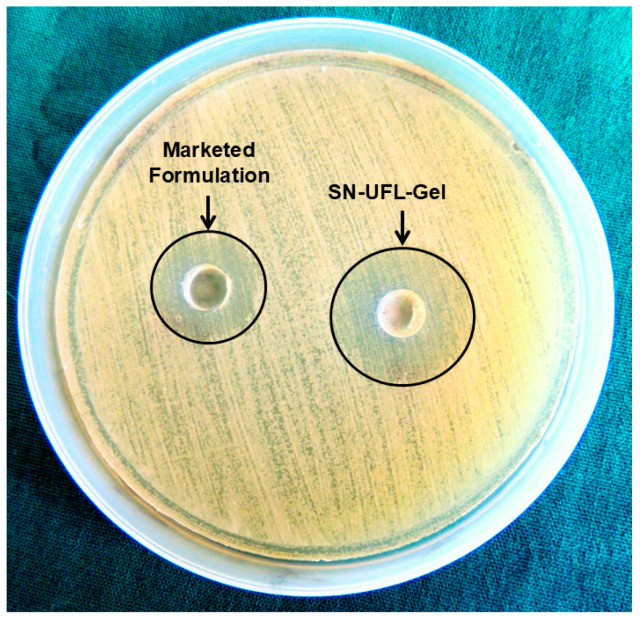
In vitro antifungal activity of SN-UFL-Gel and marketed formulation.

The in vitro antifungal activity of the SN-UFL-Gel and the marketed formulation (Onabet^®^ 2% Sertaconazole nitrate cream) was evaluated by the cup-plate method against *Candida albicans*. The SN-UFL-Gel demonstrated a larger inhibition zone (25 ± 1.50 mm) compared to the marketed formulation (20 ± 1.72 mm), indicating superior antifungal efficacy (Figure 7). This enhanced activity may be attributed to the UFL’s great flexibility and deformability, which allows it to penetrate the *Candida albicans* cell wall and block ergosterol biosynthesis, resulting in lysis of the fungal cell membrane and loss of integrity [51,52,53]. These findings align with previous reports by Singh et al. (2017), who observed that a ketoconazole transfersomal gel produced a greater inhibition zone compared to free ketoconazole gel [54].

### 2.13. In Vivo Antifungal Study

**Figure 8:**
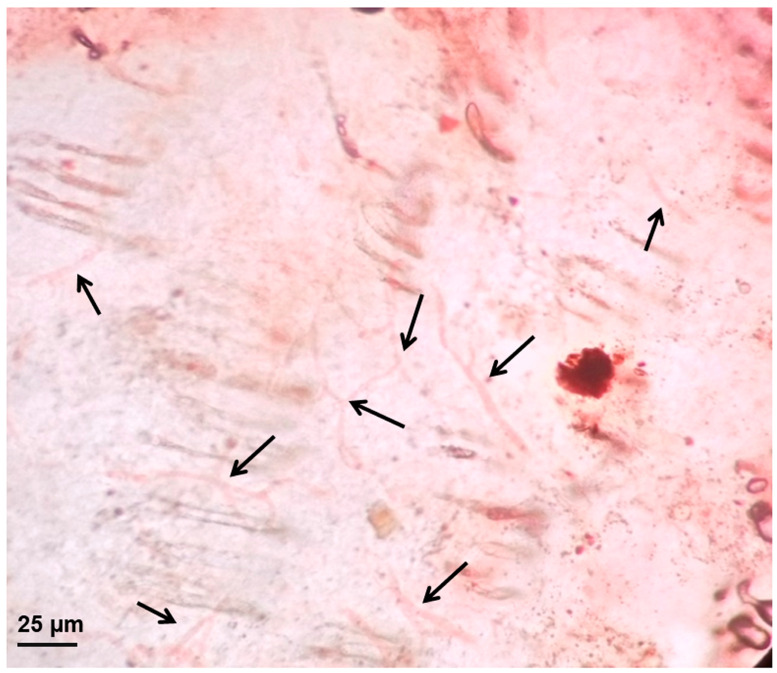
Photomicrographs confirm the induction of fungal infection on the rat skin. Black arrows represent the multiple branching fungal hyphae.

**Figure 9:**
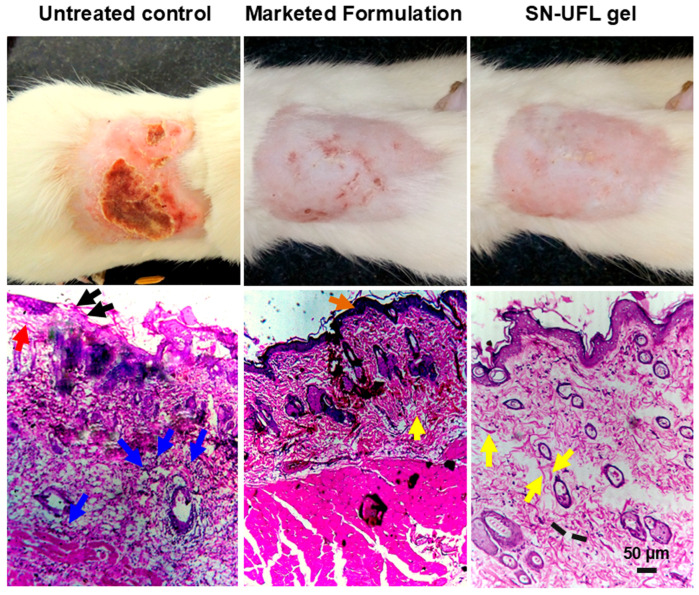
In vivo antifungal effects of untreated control, marketed formulation, and SN-UFL-Gel. Focal ulceration (red arrows), parakeratosis (black arrows), inflammatory cell infiltration in dermis (blue arrows), mild hyperkeratosis (brown arrow), fibroblast proliferation (yellow arrow), and collagen formation (black lines).

Rats were infected dorsally with the fungal strain *Trychophyton mentagrophytes*, and the characteristic symptoms of erythema and scaling were observed on the 7th day post-infection. A microscopic examination of dry skin flakes treated with 20% aqueous KOH revealed multiple branching fungal hyphae intermingled with keratinous squames and hair shafts, demonstrating active dermatophytic infection (Figure 8) [55]. Histological assessment of infected skin (untreated control, Figure 9) showed disrupted and irregular epidermis with focal ulceration (red arrows) and parakeratosis (black arrows). Intense dermal infiltration by neutrophils, lymphocytes, and macrophages, indicating both acute and chronic inflammatory responses (blue arrows). The dermis appeared to be edematous with vascular congestion and mild fibrosis. Treatment was initiated on the 8th day of infection. The lesions and wounds started to cure from the 3rd day of treatment. Rats treated with marketed cream (Onabet 2%) achieved cured on the 8th day of the treatment. Conversely, animals treated with SN-UFL-Gel were cured on the 5th day of treatment. On the 6th day of treatment, the histopathological assessment of rat skin treated with the marketed formulation showed partial restoration of normal layers in the epidermis with mild hyperkeratosis (brown arrow). The dermis showed reduced inflammatory cell infiltration, with few scattered lymphocytes and macrophages. Further dermis exhibited initial evidence of fibroblast proliferation and collagen deposition, signifying partial healing. Animals treated with SN-UFL-Gel showed a completely re-epithelialized epidermis with intact *stratum corneum* and normal epidermal architecture. The dermis appeared to be compact and less edematous, with minimal inflammatory cell infiltration. There is also prominent fibroblast activity (yellow arrow) and new collagen formation (black lines) in the dermis, demonstrating superior healing. These histopathological results indicate that the SN-UFL-Gel produced an enhanced antifungal effect, with the absence of fungal elements, restored epidermal architecture, and minimal inflammation, compared to the marketed formulation. This can be ascribed to the nano-size, deformability, and superior penetration capability of the SN-UFL-Gel, which facilitates effective drug permeation and increased drug accumulation as well as retention in skin layers [55]. Previous studies have reported that antifungal drug-loaded transfersomes are more effective than free drugs due to their deformability, which allows them to penetrate the *C. albicans* cell wall, thereby increasing the drug uptake and fungal death [21]. Furthermore, transfersomes augment antifungal effectiveness while mitigating side effects by localizing the elevated drug levels at the target location and decreasing systemic absorption [51,53].

The superior antifungal efficacy of SN-UFL-Gel observed in both in vitro and in vivo studies can be attributed to the systematic optimization process used in the study. The 3^2^ factorial designs identified the optimum quantity of phospholipid (828.78 mg) and sodium cholate (162.25 mg) that produced nanovesicles with minimum size (118.60 nm) and maximum entrapment efficiency (84.10%). The smaller vesicle size enhanced the penetration through the *stratum corneum* barrier, while higher EE delivered greater payloads of SN to the targeted site. The optimized formulation, therefore, possesses ideal characteristics required for efficient skin delivery. Previously, Abdellatif et al. (2017) carried out the in vitro and in vivo evaluation of various lipid vesicles loaded with SN [21]. However, their study did not include a systematic optimization process to determine the specific formulation variables that will impact the critical parameters, such as vesicle size and entrapment efficiency. Since optimization was not performed, the tested formulations might not represent the full potential of the carrier system. Furthermore, the incorporation of in vivo assessment utilizing a dermatophyte rat infection model offers essential proof of treatment efficacy that was lacking in prior SN-embedded flexisome studies [20]. The direct relationship between the optimization-driven design process, the physico-chemical properties of the vesicles, and the therapeutic outcomes observed in the animal model provides a solid scientific foundation for the superiority of the formulation compared to what could be achieved through optimization or in vivo testing alone. The integration of these strategies yields a more reliable and robust approach for the clinically effective topical delivery of SN.

### 2.14. Stability Studies

Investigating the stability of nanovesicular gel formulations during storage is a crucial prerequisite for their potential use as drug delivery systems. Stability tests were conducted to determine the ideal storage conditions that preserve the physical and chemical integrity of the drug product. The stability study results (Table 4) demonstrated that SN-UFL gel remained stable when subjected to various temperature settings (4 ± 2 °C, 25 ± 2 °C, and 40 ± 2 °C) for a period of 3 months. No significant alteration was seen in the pH, viscosity, and percentage of drug content. This change may result from the integration of nanovesicles into the carbopol hydrogel. The higher viscosity of Carbopol^®^ 980 NF gel will inhibit the aggregation and fusing of nanovesicles, thereby preserving their original structure and integrity. These findings corroborate earlier stability data acquired for transfersomes [36,55]. The minor reduction in drug content percentage noted at an elevated temperature (40 ± 2 °C) relative to 4 ± 2 °C and 25 ± 2 °C may be ascribed to the partial breakdown of phospholipids at this higher temperature. Therefore, it is recommended to store the formulation at a lower temperature [36,56].

### 2.15. Limitations and Future Scope

The present study has a few limitations. First, a control formulation incorporating free sertaconazole nitrate within the same carbopol gel matrix was not included. Although the superior performance of the SN-UFL-Gel over the marketed cream is evident, future work will address this issue through direct comparative evaluation with a plain SN gel. Second, while short-term stability studies (three months) confirmed the formulation’s robustness, long-term stability testing (6–24 months), along with microbial assessment, is required to establish a definitive shelf life. Finally, although the in vivo antifungal results in rodents were promising, these findings remain preclinical and warrant validation through rigorously designed clinical trials to confirm safety and efficacy in humans.

## 3. Conclusions

The current study effectively designed and optimized a phospholipid-based ultraflexible nanovesicular gel (SN-UFL-Gel) of sertaconazole nitrate via a 3^2^ factorial design to augment its topical antifungal activity. This study combines factorial design-driven formulation optimization with in vivo evaluation to ensure a robust and clinically relevant formulation. This integrated approach distinguishes it from earlier studies that did not have systematic optimization or in vivo evaluation. The optimized formulation demonstrated spherical, nano-sized vesicles characterized by high entrapment efficiency. The incorporation of SN-UFL into a carbopol gel made it easier to spread, extrude, and use by patients. Ex vivo studies revealed markedly enhanced skin permeability, deposition, and localized drug accumulation relative to the commercially available formulation, hence affirming better dermal bioavailability. The in vitro and in vivo studies proved that SN-UFL-Gel was very effective against *Candida albicans* and *Trichophyton mentagrophytes*, and it did not cause any irritation or changes in histopathology. These findings confirm ultraflexible liposomal gels as a potential nanocarrier platform for improving the therapeutic efficacy of poorly permeable antifungal agents such as sertaconazole nitrate, providing a safe and effective alternative to traditional topical preparations.

## 4. Materials and Methods

### 4.1. Materials

Sertaconazole nitrate and Phospholipon^®^ 90 G were kindly provided as gift samples by Optimus Drugs (P) Ltd. (Hyderabad, India) and Lipoid GmbH (Ludwigshafen, Germany), respectively. Sodium cholate was sourced from Sigma-Aldrich (Bengaluru, India). Analytical-grade chloroform, methanol, and ethanol were acquired from Fisher Scientific (Mumbai, India). Carbopol 980^®^ NF was supplied as a complimentary sample by Lubrizol India Private Limited (Mumbai, India). Onabet^®^ cream (2% *w*/*w* sertaconazole nitrate) was purchased from local pharmacy stores. Triethanolamine, sodium hydroxide, potassium dihydrogen phosphate, and glass beads were obtained from Himedia Laboratories Pvt. Ltd. (Mumbai, India) Sterile hydrophilic syringe filters (Minisart^®^) were obtained from Sartorius Stedim Biotech GmbH, Gottigen, Germany. Millipore water was used throughout the experiment.

### 4.2. Fungal Strains

The fungal strains *Candida albicans* (ATCC # 2091) and *Trichophyton mentagrophytes* (ATCC # 18750) were obtained from the Institute of Microbial Technology, Chandigarh, India.

### 4.3. Preparation of Sertaconazole Nitrate-Loaded Ultraflexible Liposomes (SN-UFLs)

Sertaconazole nitrate-loaded ultraflexible liposomes (SN-UFLs) were fabricated using the thin-film hydration-sonication method, adapted from the protocol established by Cevc et al. (2003) [57]. Phospholipid, sodium cholate, and SN were precisely weighed and combined in a round-bottom flask, then dissolved in a chloroform-methanol mixture (2:1 *v*/*v*). Solvent removal was achieved via rotary evaporation (Buchi R-210, Flawil, Switzerland) at 60 °C under reduced pressure, yielding a uniform lipid film. The film underwent overnight vacuum desiccation to eliminate residual solvents. Hydration was performed with pH 6.4 phosphate buffer at 60 °C (100 rpm, 1 h), resulting in a milky vesicular dispersion. The suspension was refrigerated at 4 °C for 24 h to allow complete hydration of phospholipids and stabilization of vesicle structure prior to sonication. This step ensures the formation of uniform vesicles and prevents lipid degradation. Subsequent processing involved probe sonication at 4 °C (2 min), followed by sequential filtration through sterile 0.45 µm and 0.2 µm membranes to obtain monodisperse nanovesicles. The final SN-UFL preparation was stored at 4 °C for subsequent analyses. For in vitro skin penetration studies, parallel transfersomal formulations containing rhodamine 6G (0.03% *w*/*v*) were prepared using identical procedural parameters.

### 4.4. Experimental Design

Optimization of sertaconazole nitrate-loaded ultraflexible liposomes (SN-UFLs) was conducted using a two-factor, three-level (3^2^) factorial design implemented in Design-Expert^®^ software (Version 7.0.0, Stat-Ease, Inc., Minneapolis, MN, USA). The study systematically evaluated the impact of phospholipid concentration (Phospholipon^®^ 90G, X_1_) and sodium cholate concentration (X_2_), each varied at three coded levels (–1, 0, +1), on critical quality attributes: vesicle size (Y_1_) and entrapment efficiency (Y_2_). Nine SN-UFL formulations were prepared with sertaconazole nitrate maintained at a constant concentration (1.5% *w*/*v*) across all batches [22]. Experimental design parameters, including independent variables (X_1_, X_2_) and dependent responses (Y_1_, Y_2_), are summarized in Table 1.

### 4.5. Vesicle Size, Size Distribution, and Zeta Potential

The Zetasizer Nano ZS (Malvern Instruments Ltd., Malvern, UK) was used to measure the vesicle size, size distribution, and zeta potential of SN-UFL formulations. The samples were analyzed at a temperature of 25 °C following the necessary dilution with Milli-Q water [23].

### 4.6. Entrapment Efficiency (EE)

Entrapment efficiency of all transfersomal formulations was determined by an indirect method using the ultracentrifugation technique. Two milliliters of each UFL suspension were transferred to a centrifugation tube and centrifuged at 40,000 rpm at 4 °C for 3 h in an ultracentrifuge (Sorvall™ MTX 150, Thermo Scientific, Mumbai, India). Following the centrifugation, supernatant liquid was separated from the pellet using a micropipette, and absorbance was taken at 204 nm using a UV spectrophotometer (Shimadzu-1800, Kyoto, Japan) after suitable dilutions [32]. The percentage EE was calculated using the following formula. % EE = (Total drug content − unentrapped drug in supernatant)/total drug content × 100.

### 4.7. Vesicle Morphology

A transmission electron microscope (TEM) was used to examine the morphology of the prepared nanovesicles. A drop of appropriately diluted optimized SN-UFL (OPT-SN-UFL) suspension was applied directly on the carbon-coated grid and treated with phosphotungstic acid solution (1% *w*/*v*). The samples were air-dried and observed using a TEM (H-7500, Hitachi, Tokyo, Japan) at a magnification of 100 kV [23].

### 4.8. Preparation of Sertaconazole Nitrate-Loaded Ultraflexible Liposomal Gel (SN-UFL-Gel)

A 1% *w/v* dispersion of carbopol^®^ 980NF was prepared by hydrating the polymer in deionized water under continuous agitation (500 rpm) until a transparent dispersion formed. The dispersion was equilibrated for 24 h to ensure complete polymer hydration and swelling. The optimized SN-UFL suspension (OPT-SN-UFL) was centrifuged at 40,000 rpm for 3 h at 4 °C, and the resultant pellet was reconstituted into the carbopol dispersion through mild agitation. Triethanolamine was subsequently added to adjust pH and induce gelation, yielding a homogeneous vesicle-loaded gel [49]. For comparative studies, Rh6G-loaded transfersomal suspension (Rh6G-TFS) was substituted for OPT-SN-UFL during gel preparation to formulate Rh6G-TFS-Gel.

### 4.9. Characterization of SN-UFL-Gel

#### 4.9.1. pH Evaluation

A benchtop pH meter was used to measure the pH of SN-UFL-Gel by submerging the glass electrode. The pH meter was calibrated with pH 4.0 and pH 7.0 buffers before measurement [58].

#### 4.9.2. Viscosity, Spreadability, and Extrudability

Rheological assessment of SN-UFL-Gel was conducted at 25 ± 0.5 °C using a Brookfield cone-and-plate viscometer (CAP 2000+, Brookfield Engineering Laboratories, Middleboro, MA, USA; spindle #1). A 0.1 g sample was applied to the viscometer plate beneath the spindle, with viscosity measured at 50 rpm under steady-state conditions. Spreadability was evaluated using the parallel-plate method: 0.5 g of gel was placed within a 2 cm diameter circle on a glass plate (75 × 25 × 1 mm), covered with a second plate, and compressed under a 0.5 kg load for 5 min. The percentage spread by area was calculated as % Spreadability = (Final area/Initial area) × 100. Extrudability was quantified as the force required to expel ≥ 0.5 cm of gel ribbon within 10 s from an aluminum tube, expressed as: Extrudability (g/cm^2^) = Applied weight (g)/Cross-sectional area (cm^2^) [22].

#### 4.9.3. Drug Content

An accurately weighed quantity of the SN-UFL-Gel (0.2 g) was dissolved in 20 mL of methanol. For complete solubilization of the drug, the solution was stirred at 700 rpm for 10 min and sonicated for 5 min. Finally, the solution was passed through 0.45 µm filters, suitably diluted with PBS, and analyzed by using a UV-Vis spectrophotometer at 204 nm to estimate the amount of SN present in the gel. The drug content is indicated in terms of percentage [22].

### 4.10. In Vitro Skin Permeation and Deposition Studies

In vitro skin permeation studies were conducted using full-thickness abdominal skin harvested from male Wistar rats. All animal protocols were approved by the Institutional Animal Ethics Committee of KLE College of Pharmacy, Belagavi (Ref: KLECOP/IAEC/Res.22–10/05/2015). After epilation using electric clippers and depilatory cream, the subcutaneous adipose layer was carefully excised. The skin was rinsed with phosphate-buffered saline (PBS) pH 6.8, sectioned to fit Franz diffusion cells (PermeGear, Inc. (Hellertown, PA, USA); effective diffusion area: 1.77 cm^2^; receptor volume: 12 mL), and mounted with the *stratum corneum* oriented toward the donor compartment. The receptor medium (PBS: ethanol 50:50 *v*/*v*) was maintained at 37 ± 0.5 °C with continuous stirring (100 rpm) to ensure sink conditions. Appropriate quantities of SN-UFL-Gel and marketed Onabet^®^ cream were applied topically to the donor compartment. Receptor samples (0.5 mL) were collected at 1, 2, 3, 4, 6, 8, 10, and 12 h intervals, with immediate replacement by fresh receptor medium. Drug quantification was performed via UV spectrophotometry (Shimadzu UV-1800; 204 nm) after appropriate dilution. Permeation profiles were generated by plotting cumulative drug permeated (μg/cm^2^) versus time, and permeation parameters like flux (J) and permeability coefficient (K) values were calculated using the formula reported in the literature [59]. All the values were expressed as the mean ± standard deviation of six independent experiments (n = 6). Statistical significance between the SN-UFL-Gel and the marketed formulation was determined using an unpaired Student *t*-test using GraphPad Prism (version 8.0.2) software.

Following in vitro permeation studies, the skin was carefully excised from Franz diffusion cells for drug deposition analysis in skin layers. Residual formulation was removed by methanol rinsing, followed by tissue processing: skin sections were minced, homogenized in methanol (1:5 *w*/*v*), and subjected to probe sonication (4 °C, 60 min) for complete drug extraction. The homogenate was centrifuged (10,000 rpm, 10 min), filtered through 0.45 µm membranes, and analyzed for SN content via UV spectrophotometry (204 nm). The local accumulation efficiency (LAC) values for SN were computed using the following equation [10].

LAC = Quantity of SN deposited in the skin after 12 h/Cumulative amount of SN permeated after 12 h.

### 4.11. Skin Penetration Study

Fluorescence microscopy was employed to evaluate the skin penetration capability of transfersomes. Full-thickness abdominal skin from male Wistar rats (prepared for in vitro permeation protocol) was mounted in Franz diffusion cells with the *stratum corneum* oriented toward the donor compartment. The receptor chamber was filled with PBS/ethanol (50:50 *v*/*v*) and maintained at 37 ± 0.5 °C with continuous agitation (100 rpm). Rhodamine-6G-loaded transfersomal gel (Rh6G-TFS-Gel) was applied non-occlusively to the epidermal surface for 8 h under physiological conditions. Post-exposure, the skin was excised, rinsed with deionized water to remove residual formulation, and fixed in 10% buffered formalin. Tissue sections were paraffin-embedded, microtomed (5 μm), mounted on glass slides, and visualized under fluorescence microscopy (excitation 530 nm, emission 580 nm). Rhodamine-6G plain gel (Rh6G-Gel) served as the negative control for comparative analysis [60].

### 4.12. Skin Irritation Studies

A skin irritation study was conducted using male Wistar rats (150–180 g) following OECD Guideline 404. Dorsal hair was removed using electric clippers (proximal to distal direction) without epidermal compromise. Animals were randomized into four groups (n = 6): Group 1 (vehicle control), Group 2 (placebo UFL gel), Group 3 (SN-UFL-Gel), and Group 4 (marketed formulation). Topical application (0.5 g) was administered daily for 7 days to a 2.5 cm^2^ shaved dorsal region. Treated sites were monitored daily for erythema and edema. Post-study, animals were euthanized, and skin samples (2 × 2 cm) were excised, fixed in 10% neutral buffered formalin, embedded in wax, and processed for histopathology. Five-micrometer sections were stained with hematoxylin and eosin (H&E) and evaluated via light microscopy for epidermal integrity, inflammatory infiltration, and dermal morphology [61].

### 4.13. In Vitro Antifungal Activity

The in vitro antifungal efficacy of the SN-UFL-Gel was carried out by the cup plate method with *Candida albicans* (ATCC # 2091). A sterilized sabouraud dextrose agar (SDA) medium was poured into sterile Petri dishes in an aseptic environment and allowed to solidify. The aqueous suspension of *C. albicans* was uniformly spread on the solidified SDA media. Two wells or cups were made using a sterile borer, which were filled with SN-UFL-Gel and the marketed formulation, respectively, using sterile syringes. The plates were covered with a lid and incubated at 37 °C for 24 h. The zone of inhibition (mm) was measured for both the formulations [51].

### 4.14. In Vivo Antifungal Activity

All procedures received prior approval from the Institutional Animal Ethics Committee (IAEC) of KLE College of Pharmacy, Belagavi (Ref: KLECOP/IAEC/Res.22-10/2015) and adhered to the Committee for the Purpose of Control and Supervision of Experiments on Animals (CPCSEA) guidelines. Male Wistar rats (200–250 g; n = 6/group) were randomized into three cohorts: Group I (untreated control), Group II (infected + Onabet^®^ cream), and Group III (infected + SN-UFL-Gel). The sample size (n = 6 per group) was determined based on similar studies reported in the literature and considerations of statistical power. This number is frequently used in preclinical dermatological investigations to identify statistically significant disparities among treatment groups, while adhering to the principles of reduction in animal use. Fungal infection was induced by shaving a 4 cm^2^ dorsal region, gently abrading the epidermis with sterile 100-grit sandpaper, and applying 200 µL of *Trichophyton mentagrophytes* inoculum (1 × 10^6^ CFU/mL). Infection was confirmed 7 days post-inoculation via KOH microscopy of skin scrapings. To ensure dose uniformity, the amount of sertaconazole nitrate (SN) applied per treatment was standardized across all groups. Each animal received a daily topical dose of 0.5 g of the marketed formulation and 0.67 g of SN-UFL-Gel, which contains 10 mg equivalent of SN. Topical treatment for 6 days began after hyphal confirmation, with daily clinical evaluation for erythema, scaling, and lesion regression. Post-treatment, animals were euthanized, and full-thickness skin biopsies were harvested, fixed in 10% neutral buffered formalin, processed for histopathology, and evaluated for epidermal integrity and fungal clearance [46].

### 4.15. Stability Studies

The stability of SN-UFL-Gel was carried out in aluminum collapsible tubes maintained at three distinct temperatures: 4 ± 2 °C, 25 ± 2 °C, and 40 ± 2 °C for a duration of three months. The gels were assessed for pH, viscosity, and % drug content at the end of the storage time [36].

## Figures and Tables

**Table 1:** 3^2^ factorial design summarizing the independent and dependent variables along with their observed responses, PDI, and ZP of SN-UFL formulations.

Formulation Code	Factor X_1_	Factor X_2_	Y_1_: Vesicle Size (nm)	Y_2_: % EE	PDI	ZP (mV)
SN-UFL 1	−1	−1	118.10 ± 1.20	79.50 ± 1.08	0.18 ± 0.01	−21.5 ± 1.45
SN-UFL 2	−1	0	114.32 ± 1.52	81.68 ± 1.50	0.20 ± 0.02	−26.7 ± 1.80
SN-UFL 3	−1	1	104.45 ± 0.90	77.60 ± 0.85	0.15 ± 0.01	−42.2 ± 2.54
SN-UFL 4	0	−1	129.30 ± 1.54	82.57 ± 1.20	0.21 ± 0.01	−29.7 ± 1.95
SN-UFL 5	0	0	121.25 ± 1.95	85.60 ± 1.10	0.23 ± 0.02	−35.2 ± 2.10
SN-UFL 6	0	1	111.42 ± 1.10	80.80 ± 1.50	0.27 ± 0.03	−39.8 ± 2.24
SN-UFL 7	1	−1	151.98 ± 2.50	83.02 ± 1.84	0.24 ± 0.01	−39.1 ± 2.40
SN-UFL 8	1	0	146.52 ± 2.14	86.04 ± 1.90	0.27 ± 0.02	−44.3 ± 2.80
SN-UFL 9	1	1	139.30 ± 1.80	82.57 ± 1.05	0.28 ± 0.02	−51.2 ± 2.45
Factors				Levels used, actual (coded)		
Independent variables				Low (−1)	Medium (0)	High (+1)
X_1_: Phospholipon 90G (mg) X_2_: Sodium cholate (mg)				750	850	950
				50	150	250
Dependent variables						
Y_1_: Vesicle size (nm)				Minimize		
Y_2_: Entrapment efficiency (%)				Maximize		

PDI: polydispersity index, ZP: zeta potential.

**Table 2:** Model summary statistics for responses Y_1_ (vesicle size) and Y_2_ (% entrapment efficiency) of SN-UFLs for fitting into different models.

Models	SD	R^2^	Adjusted R^2^	Predicted R^2^	PRESS	CV (%)	Remark
Response (Y_1_)							
Linear	5.13	0.9276	0.9035	0.8555	314.95	4.06	
2FI	5.61	0.9277	0.8844	0.7818	475.74	4.05	
Quadratic	1.79	0.9956	0.9882	0.9474	114.66	1.42	Suggested
Response (Y_2_)							
Linear	2.14	0.5241	0.3654	0.0079	57.46	2.61	
2FI	2.33	0.5331	0.2530	−1.0995	121.59	2.83	
Quadratic	0.35	0.9938	0.9835	0.9440	3.24	0.42	Suggested

SD: Standard deviation, R^2^: multiple correlation coefficient, 2FI: Two-factor interaction, PRESS: Predicted residual sum of squares, CV: Coefficient of Variation.

**Table 3:** ANOVA Results and adequate precision values for each response of SN-UFL.

Source	Responses					
	Y_1_ (Size)			Y_2_ (Entrapment Efficiency %)		
	F-Value	*p*-Value Prob > F	Adequacy Precision	F-Value	*p*-Value Prob > F	Adequacy Precision
Model	135.32	0.0010	33.073	96.56	0.0016	31.082
X_1_	528.92	0.0002		230.85	0.0006	
X_2_	101.50	0.0021		23.73	0.0165	
X_1_X_2_	0.094	0.7789		4.41	0.1266	
X_1_^2^	44.51	0.0069		26.42	0.0143	
X_2_^2^	1.60	0.2957		197.37	0.0008	

**Table 4:** Stability of SN-UFL-Gel at different storage conditions.

Time	Storage Condition								
	4 ± 2 °C			25 ± 2 °C			40 ± 2 °C		
	pH	Viscosity	% Drug Content	pH	Viscosity	% Drug Content	pH	Viscosity	% Drug Content
Initial	6.50 ± 0.01	499.66 ± 15.10	96.70 ± 0.20	6.50 ± 0.01	499.66 ± 15.10	96.70 ± 0.20	6.50 ± 0.02	499.66 ± 15.10	96.70 ± 0.20
Three months	6.48 ± 0.02	497.35 ± 18.35	96.10 ± 0.17	6.42 ± 0.01	492.10 ± 18.20	94.28 ± 0.10	6.45 ± 0.02	489.15 ± 20.45	90.54 ± 0.68

## Data Availability

The raw data are available from the corresponding author upon reasonable request.

## Abbreviations

The following abbreviations are used in this manuscript:

SN	Sertaconazole nitrate
SN-UFL	Sertaconazole nitrate-loaded ultraflexible liposomes
ZP	Zeta potential
EE	Entrapment efficiency
OPT-SN-UFL	Optimized Sertaconazole nitrate-loaded ultraflexible liposomes
AIDS	Acquired Immune Deficiency Syndrome
R^2^	Coefficient of correlation
PRESS	Predicted residual error sum of squares
% CV	Percentage coefficient of variation
PDI	Polydispersity index
SD	Standard deviation
ANOVA	Analysis of variance
3D	Three dimensional
2D	Two dimensional
LAC	Local accumulation efficacy
Rh6G	Rhodamine-6G
Rh6G-TFS-Gel	Rhodamine-6G-loaded transfersomal gel
SC	Stratum corneum
OECD	Organization for economic co-operation and development
KOH	Potassium hydroxide
CPCSEA	Committee for the Purpose of Control and Supervision of Experiments on Animals

## References

[1] Urban K., Chu S., Scheufele C., Giesey R.L., Mehrmal S., Uppal P., Delost G.R. (2020). The global, regional, and national burden of fungal skin diseases in 195 countries and territories: A cross-sectional analysis from the Global Burden of Disease Study 2017. JAAD Int..

[2] Garg A., Sharma G.S., Goyal A.K., Ghosh G., Si S.C., Rath G. (2020). Recent advances in topical carriers of antifungal agents. Heliyon.

[3] Walsh T.J., Dixon D.M., Baron S. (1995). Spectrum of mycoses. Medical Microbiology.

[4] Aditya K.G., Jennifer E.R., Melody C., Elizabeth A.C. (2005). Dermatophytosis: The management of fungal infections. Ski. Dermatol. Clin..

[5] Akhtar N., Verma A., Pathak K. (2015). Topical delivery of drugs for the effective treatment of fungal infections of skin. Curr. Pharm. Des..

[6] Sahni K., Singh S., Dogra S. (2018). Newer topical treatments in skin and nail dermatophyte infections. Indian Dermatol. Online J..

[7] Pfaller M.A., Sutton D.A. (2006). Review of in vitro activity of sertaconazole nitrate in the treatment of superficial fungal infections. Diagn. Microbiol. Infect. Dis..

[8] Carrillo-Muñoz A.J., Tur-Tur C., Giusiano G., Marcos-Arias C., Eraso E., Jauregizar N., Quindós G. (2013). Sertaconazole: An antifungal agent for the topical treatment of superficial candidiasis. Expert Rev. Anti-Infect. Ther..

[9] Liebel F., Lyte P., Garay M., Babad J., Southall M.D. (2006). Anti-inflammatory and anti-itch activity of sertaconazole nitrate. Arch. Dermatol. Res..

[10] Younes N.F., Habib B.A. (2022). Augmented local skin accumulation efficiency of sertaconazole nitrate via glycerosomal hydrogel: Formulation, statistical optimization, ex vivo performance and in vivo penetration. J. Drug Deliv. Sci. Technol..

[11] Chacko I.A., Ghate V.M., Dsouza L., Lewis S.A. (2020). Lipid vesicles: A versatile drug delivery platform for dermal and transdermal applications. Colloids Surf. B Biointerfaces.

[12] Opatha S.A.T., Titapiwatanakun V., Chutoprapat R. (2020). Transfersomes: A promising nanoencapsulation technique for transdermal drug delivery. Pharmaceutics.

[13] Soliman G.M., Attia M.A., Mohamed R.A. (2014). Poly(ethylene glycol)-block-poly(ε-caprolactone) nanomicelles for the solubilization and enhancement of antifungal activity of sertaconazole. Curr. Drug Deliv..

[14] Pande V.V., Kadnor N.A., Kadam R.N., Upadhye S.A. (2015). Fabrication and characterization of sertaconazole nitrate microsponge as a topical drug delivery system. Indian J. Pharm. Sci..

[15] Manian M., Madrasi K., Chaturvedula A., Banga A.K. (2016). Investigation of the dermal absorption and irritation potential of sertaconazole nitrate anhydrous gel. Pharmaceutics.

[16] Patil M., Bhagade P., Amale M., Sonawane S., Kshirsagar S. (2021). Development of sertaconazole nitrate loaded nanostructured lipid carriers gel using central composite design: In vitro and Ex vivo evaluation. Nanosci. Nanotechnol. Asia.

[17] Tavakoli N., Taymouri S., Saeidi A., Akbari V. (2019). Thermosensitive hydrogel containing sertaconazole loaded nanostructured lipid carriers for potential treatment of fungal keratitis. Pharm. Dev. Technol..

[18] Abdellatif M.M., Khalil I.A., Elakkad Y.E., Eliwa H.A., Samir T.M., Al-Mokaddem A.K. (2020). Formulation and characterization of sertaconazole nitrate mucoadhesive liposomes for vaginal candidiasis. Int. J. Nanomed..

[19] Abdellatif M.M., Josef M., El-Nabarawi M.A., Teaima M. (2022). Sertaconazole-nitrate-loaded leciplex for treating keratomycosis: Optimization using D-optimal design and in vitro, ex vivo, and in vivo studies. Pharmaceutics.

[20] Mandlik S.K., Siras S.S., Birajdar K.R. (2019). Optimization and characterization of sertaconazole nitrate flexisomes embedded in hydrogel for improved antifungal activity. J. Liposome Res..

[21] Abdellatif M.M., Khalil I.A., Khalil M.A. (2017). Sertaconazole nitrate loaded nanovesicular systems for targeting skin fungal infection: In-vitro, ex-vivo and in-vivo evaluation. Int. J. Pharm..

[22] Kanshide A., Peram M.R., Chandrasekhar N., Jamadar A., Kumbar V., Kugaji M. (2023). Formulation, optimization, and antioxidant evaluation of tetrahydrocurcumin-loaded ultradeformable nanovesicular cream. J. Pharm. Innov..

[23] Jamadar A.T., Peram M.R., Chandrasekhar N., Kanshide A., Kumbar V.M., Diwan P.V. (2023). Formulation, optimization, and evaluation of ultradeformable nanovesicles for effective topical delivery of hydroquinone. J. Pharm. Innov..

[24] Verma D.D., Verma S., Blume G., Fahr A. (2003). Particle size of liposomes influences dermal delivery of substances into skin. Int. J. Pharm..

[25] Akombaetwa N., Ilangala A.B., Thom L., Memvanga P.B., Witika B.A., Buya A.B. (2023). Current advances in lipid nanosystems intended for topical and transdermal drug delivery applications. Pharmaceutics.

[26] Arora D., Khurana B., Nanda S. (2020). Statistical development and in vivo evaluation of resveratrol-loaded topical gel containing deformable vesicles for a significant reduction in photo-induced skin aging and oxidative stress. Drug Dev. Ind. Pharm..

[27] Ahad A., Aqil M., Kohli K., Sultana Y., Mujeeb M., Ali A. (2012). Formulation and optimization of nanotransfersomes using experimental design technique for accentuated transdermal delivery of valsartan. Nanomed. Nanotechnol. Biol. Med..

[28] Khatoon K., Rizwanullah M.D., Amin S., Mir S.R., Akhter S. (2019). Cilnidipine loaded transfersomes for transdermal application: Formulation optimization, in-vitro and in-vivo study. J. Drug Deliv. Sci. Technol..

[29] Shreya A.B., Managuli R.S., Menon J., Kondapalli L., Hegde A.R., Avadhani K., Shetty P.K., Amirthalingam M., Kalthur G., Mutalik S. (2016). Nano-transfersomal formulations for transdermal delivery of asenapine maleate: In vitro and in vivo performance evaluations. J. Liposome Res..

[30] Zhang S., Han X., Yang L., Liang W., Xu H. (2016). Effect of vesicle-to-micelle transition on the interactions of phospholipid/sodium cholate mixed vesicles. J. Phys. Chem. B.

[31] Motawea A., Maria S.N., Maria D.N., Jablonski M.M., Ibrahim M.M. (2024). Genistein transfersome-embedded topical delivery system for skin melanoma treatment: In vitro and ex vivo evaluations. Drug Deliv..

[32] Peram M.R., Suryadevara V., Patil S., Kunam V., Kumbar V., Babar P., Galatage S., Arehalli M. (2025). Development of curcumin-loaded ultradeformable lipid vesicles for enhanced anti-melanoma activity: In vitro, ex vivo, and cell line studies. J. Dispers. Sci. Technol..

[33] Ali M.F., Salem H.F., Abdelmohsen H.F., Attia S.K. (2015). Preparation and clinical evaluation of nano-transferosomes for treatment of erectile dysfunction. Drug Des. Dev. Ther..

[34] Albash R., Elmahboub Y., Baraka K., Abdellatif M.M., Alaa-Eldin A.A. (2020). Ultra-deformable liposomes containing terpenes (terpesomes) loaded with fenticonazole nitrate for treatment of vaginal candidiasis: Box–Behnken design optimization, comparative ex vivo and in vivo studies. Drug Deliv..

[35] Slavkova M., Tzankov B., Popova T., Voycheva C. (2023). Gel Formulations for Topical Treatment of Skin Cancer: A Review. Gels.

[36] Opatha S.A., Titapiwatanakun V., Boonpisutiinant K., Chutoprapat R. (2022). Preparation, Characterization and Permeation Study of Topical Gel Loaded with Transfersomes Containing Asiatic Acid. Molecules.

[37] Khan M.A., Iqbal Z., Aqil M. (2024). Carbopol 934-Based Transethosomal Gel of Glycyrrhizic Acid for the Management of Skin Cancer. J. Drug Deliv. Sci. Technol..

[38] Raut S.S., Rane B.R., Jain A.S. (2023). Development and Evaluation of Ebastine-Loaded Transfersomal Nanogel for the Treatment of Urticaria (Autoimmune Disease). Eng. Proc..

[39] Neupane R., Boddu S.H., Renukuntla J., Babu R.J., Tiwari A.K. (2020). Alternatives to Biological Skin in Permeation Studies: Current Trends and Possibilities. Pharmaceutics.

[40] Ainbinder D., Touitou E. (2005). Testosterone Ethosomes for Enhanced Transdermal Delivery. Drug Deliv..

[41] Ghazwani M., Alqarni M.H., Hani U., Alam A. (2023). QbD-Optimized, Phospholipid-Based Elastic Nanovesicles for the Effective Delivery of 6-Gingerol: A Promising Topical Option for Pain-Related Disorders. Int. J. Mol. Sci..

[42] Demartis S., Rassu G., Murgia S., Casula L., Giunchedi P., Gavini E. (2021). Improving Dermal Delivery of Rose Bengal by Deformable Lipid Nanovesicles for Topical Treatment of Melanoma. Mol. Pharm..

[43] Morilla M.J., Romero E.L. (2015). Ultradeformable Phospholipid Vesicles as a Drug Delivery System: A Review. Res. Rep. Transdermal Drug Deliv..

[44] Albratty M. (2023). Design, Optimization, and Characterization of *Althaea officinalis*-Loaded Transliposomes for the Treatment of Atopic Dermatitis: A Box–Behnken Design, In Vitro, and Ex Vivo Study. J. Biomater. Sci. Polym. Ed..

[45] Ma M., Wang J., Guo F., Lei M., Tan F., Li N. (2015). Development of Nanovesicular Systems for Dermal Imiquimod Delivery: Physicochemical Characterization and In Vitro/In Vivo Evaluation. J. Mater. Sci. Mater. Med..

[46] Guo F., Wang J., Ma M., Tan F., Li N. (2015). Skin Targeted Lipid Vesicles as Novel Nano-Carrier of Ketoconazole: Characterization, In Vitro and In Vivo Evaluation. J. Mater. Sci. Mater. Med..

[47] Chen J., Lu W.L., Gu W., Lu S.S., Chen Z.P., Cai B.C. (2013). Skin Permeation Behavior of Elastic Liposomes: Role of Formulation Ingredients. Expert Opin. Drug Deliv..

[48] Alam P., Imran M., Gupta D.K., Akhtar A. (2023). Formulation of Transliposomal Nanocarrier Gel Containing Strychnine for the Effective Management of Skin Cancer. Gels.

[49] Hussain A., Samad A., Ramzan M., Ahsan M.N., Rehman Z.U., Ahmad F.J. (2016). Elastic Liposome-Based Gel for Topical Delivery of 5-Fluorouracil: In Vitro and In Vivo Investigation. Drug Deliv..

[50] Salem H.F., Nafady M.M., Kharshoum R.M., Abd El-Ghafar O.A., Farouk H.O. (2020). Mitigation of Rheumatic Arthritis in a Rat Model via Transdermal Delivery of Dapoxetine HCl Amalgamated as a Nanoplatform: In Vitro and In Vivo Assessment. Int. J. Nanomed..

[51] Alyahya E.M., Alwabsi K., Aljohani A.E., Albalawi R., El-Sherbiny M., Ahmed R., Mortagi Y., Qushawy M. (2023). Preparation and Optimization of Itraconazole Transferosomes-Loaded HPMC Hydrogel for Enhancing Its Antifungal Activity: 2^3^ Full Factorial Design. Polymers.

[52] Qushawy M., Nasr A., Abd-Alhaseeb M., Swidan S. (2018). Design, Optimization and Characterization of a Transfersomal Gel Using Miconazole Nitrate for the Treatment of Candida Skin Infections. Pharmaceutics.

[53] Hady M.A., Darwish A.B., Abdel-Aziz M.S., Sayed O.M. (2022). Design of Transfersomal Nanocarriers of Nystatin for Combating Vulvovaginal Candidiasis: A Different Perspective. Colloids Surf. B Biointerfaces.

[54] Singh S., Verma D., Mirza M.A., Das A.K., Anwer M.K., Sultana Y., Talegaonkar S., Iqbal Z. (2017). Development and Optimization of Ketoconazole-Loaded Nano-Transfersomal Gel for Vaginal Delivery Using Box–Behnken Design: In Vitro, Ex Vivo Characterization and Antimicrobial Evaluation. J. Drug Deliv. Sci. Technol..

[55] Aggarwal N., Goindi S. (2012). Preparation and Evaluation of Antifungal Efficacy of Griseofulvin-Loaded Deformable Membrane Vesicles in an Optimized Guinea Pig Model of *Microsporum canis*—Dermatophytosis. Int. J. Pharm..

[56] Chaudhary H., Kohli K., Kumar V. (2014). A Novel Nano-Carrier Transdermal Gel Against Inflammation. Int. J. Pharm..

[57] Cevc G., Gebauer D., Stieber J., Schätzlein A., Blume G. (1998). Ultraflexible Vesicles, Transfersomes, Have an Extremely Low Pore Penetration Resistance and Transport Therapeutic Amounts of Insulin across the Intact Mammalian Skin. Biochim. Biophys. Acta Biomembr..

[58] Vasanth S., Dubey A., Ravi G.S., Lewis S.A., Ghate V.M., El-Zahaby S.A., Hebbar S. (2020). Development and Investigation of Vitamin C-Enriched Adapalene-Loaded Transfersome Gel: A Collegial Approach for the Treatment of Acne Vulgaris. AAPS J..

[59] Peram M.R., Jalalpure S., Kumbar V., Patil S., Joshi S., Bhat K., Diwan P. (2019). Factorial Design-Based Curcumin Ethosomal Nanocarriers for Skin Cancer Delivery: In Vitro Evaluation. J. Liposome Res..

[60] Yusuf M., Sharma V., Pathak K. (2014). Nanovesicles for Transdermal Delivery of Felodipine: Development, Characterization, and Pharmacokinetics. Int. J. Pharm. Investig..

[61] Peram M.R., Dhananjay C., Chandrasekhar N., Kumbar V.M., Suryadevara V., Patil S.R., El-Zahaby S.A. (2024). Acitretin-Loaded Nanoethosomal Gel for the Treatment of Psoriasis: Formulation, Optimization, In Vitro and In Vivo Assessment. J. Dispers. Sci. Technol..

