# Correction: Peram et al. Phospholipid-Based Ultraflexible Nanovesicular Gel of Sertaconazole Nitrate for the Treatment of Skin Fungal Infections: Statistical Optimization, In Vitro and Preclinical Assessment. *Gels* 2025, *11*, 909

**DOI:** 10.3390/gels12040275

**Published:** 2026-03-26

**Authors:** Malleswara Rao Peram, Sachin R. Patil, Vidyadhara Suryadevara, Srinivasa Rao Yarguntla, Smita Kamalakar, Preeti Patil, Kamala Kumari Paravastu, Anand Vishwas Deshmukh, Manohar Kugaji, Sameer Nadaf

**Affiliations:** 1 Department of Pharmaceutics, Chebrolu Hanumaiah Institute of Pharmaceutical Sciences, Guntur 522019, Andhra Pradesh, India; svidyadhara@gmail.com; 2 Department of Pharmaceutics, Sarojini College of Pharmacy, Kolhapur 416004, Maharashtra, India; smitakamalakar3@gmail.com (S.K.); pbp.scop@gmail.com (P.P.); 3 Department of Pharmaceutics, Vignan Institute of Pharmaceutical Technology, Visakhapatnam 530049, Andhra Pradesh, India; ysrvignan@gmail.com (S.R.Y.); kamalaparavastu@gmail.com (K.K.P.); 4 Department of Pharmaceutics, KLE College of Pharmacy, KLE Academy of Higher Education and Research, Belagavi 590010, Karnataka, India; deshmukhanand7@gmail.com; 5 Central Research Laboratory, Maratha Mandal’s Nathajirao G. Halgekar Institute of Dental Sciences & Research Centre, Belagavi 590010, Karnataka, India; manoharkugaji@gmail.com; 6 Department of Pharmaceutics, Bharati Vidyapeeth College of Pharmacy, Palus 416310, Maharashtra, India; sam.nadaf@rediffmail.com

The author Anand Vishwas Deshmukh was not included as an author in the original publication [1]. The corrected Author Contributions statement appears here. The authors state that the scientific conclusions are unaffected. This correction was approved by the Academic Editor. The original publication has also been updated.
